# Primary Malignant Melanoma of the Mandible: A Diagnostic Dilemma and Surgical Management of a Rare Entity

**DOI:** 10.7759/cureus.108290

**Published:** 2026-05-05

**Authors:** Sanjay Soni, Nikhil Arora, Ridhima Malik, Ditixa Patel, Ramanuj Bansal, Sabareesh M, Harish Taneja

**Affiliations:** 1 Otolaryngology-Head and Neck Surgery, Maulana Azad Medical College, New Delhi, IND

**Keywords:** head and neck malignancy, malignant melanoma, mandibular melanoma, oral melanoma, pigmented oral lesion

## Abstract

Oral malignant melanoma (OMM) is a rare but highly aggressive malignancy that develops in the mucosal lining of the oral cavity. Of all primary melanoma cases, those arising from the oral cavity are extremely uncommon, and among these, involvement of the mandible as the primary site is even rarer. A 42-year-old woman presented with a bluish pigmented lesion over the left mandibular gingiva, associated with mild pain and intermittent bleeding from the lesion. Clinical examination revealed a melanocytic lesion involving the gingiva. Imaging studies, including contrast-enhanced computed tomography (CECT) and positron emission tomography (PET), revealed a metabolically active lesion with bone involvement. A biopsy confirmed the diagnosis of oral malignant melanoma. The patient underwent wide local excision with segmental mandibulectomy and elective neck dissection with pectoralis major myocutaneous flap reconstruction and adjuvant radiotherapy. The postoperative period was satisfactory. The patient remains disease-free at 18-month follow-up, with no signs of recurrence. OMM, though rare, requires high clinical suspicion due to its resemblance to other benign lesions. Early detection and aggressive surgical resection remain the key to a favorable outcome. Due to its rarity, it is not uncommon to mistake this oral melanotic lesion for other benign oral lesions such as dental amalgam, drug-induced pigmentation, Peutz-Jeghers syndrome, and even Addison's disease. The prognosis remains poor due to its aggressive nature and tendency for early metastasis. This case emphasizes the importance of timely diagnosis and multidisciplinary treatment for this rare malignancy.

## Introduction

Oral malignant melanoma (OMM) is a sporadic and aggressive form of melanoma that originates in the oral mucosal lining. It is characterized by uncontrolled proliferation of melanocytes, the cells derived from the neural crest. Although melanoma is less common than basal and squamous cell carcinoma, it accounts for the majority of skin cancer-related mortality. It constitutes about 3% to 5% of all cutaneous malignant lesions [1]. However, less than 1% of all primary melanomas originate in the oral cavity [2], making it an exceptionally uncommon oral malignant tumor. Compared with the rest of the world, oral melanomas are relatively more common in countries such as Uganda, Japan, and India [3].

Patients are either asymptomatic or present with variable symptoms such as bleeding, pain, or swelling in the mouth. The most common sites of occurrence are the hard palate and maxillary gingiva (80%), while the rest of the oral cavity is less commonly involved [4]. Involvement of the mandible is an exceedingly unique presentation. Histologically, OMM may appear as a deeply pigmented lesion, but the clinical and radiological features can mimic various benign conditions, such as oral melanotic macules or other oral mucosal lesions. The lack of clinical specificity makes early detection challenging. Clinical red flags for pigmented oral lesions include a recent-onset lesion that is increasing in size, showing asymmetry and irregular borders, and unexplained bleeding. Associated features like pain, paraesthesia, and loosening of the tooth should always alert the clinician to biopsy.

Owing to the rarity of the disease, there is a lack of conclusive evidence on the exact etiopathogenesis and risk factors. The treatment of OMM includes an aggressive surgical resection with wide margins, followed by adjuvant radiotherapy. However, in metastatic disease, chemotherapy or immunotherapy may be added. This article describes a case of oral malignant melanoma arising from the mandible, highlighting its clinical features, diagnostic workup, treatment options, and prognosis, in the context of the existing literature on this rare malignancy.

## Case presentation

A 42-year-old woman presented with complaints of a pigmented lesion in the left mandibular molar area, which had appeared about six months ago. The lesion was associated with mild pain and episodes of bleeding during chewing food or while brushing. There was no history of recent trauma, dental extraction, lower lip numbness, previous denture use, or any substance abuse.

Intraoral examination revealed a hyperplastic melanocytic lesion, approximately 1 cm in diameter, located in the attached mandibular gingiva near the left first molar region (Figure 1). On palpation, the lesion was firm and appeared attached to the underlying bone. There were no other visible melanocytic lesions in the oral cavity. The neck examination was unremarkable, with no visible or palpable swelling. Her past medical history was insignificant, and there was no family history of similar lesions.

**Figure 1:**
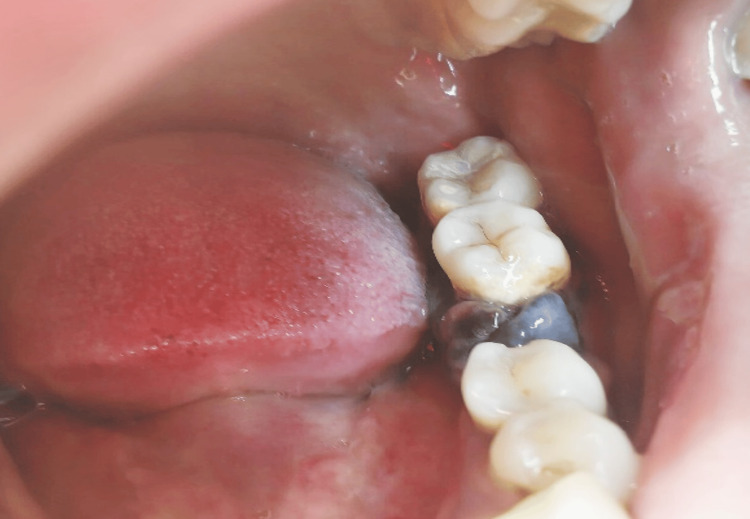
Hyperplastic melanocytic lesion involving the first mandibular molar gingiva (preoperative picture).

A contrast-enhanced computed tomography (CECT) scan of the oral cavity showed a 10 x 8 mm soft tissue lesion in the empty molar fossa of the left lower first molar (Figure 2), with lytic erosion of the adjoining mandibular alveolus. A CECT of the chest was done, which was normal. To exclude metastasis, a positron emission tomography (PET-CECT) scan was done, which suggested a metabolically active lesion involving the left mandibular first molar gingiva (SUVmax-17.6), and there was no evidence of any metabolically active lesions elsewhere in the body (Figure 3). According to the eighth edition of the American Joint Committee on Cancer staging system for head and neck mucosal melanoma, the tumor was staged as T4aN0M0. 

**Figure 2:**
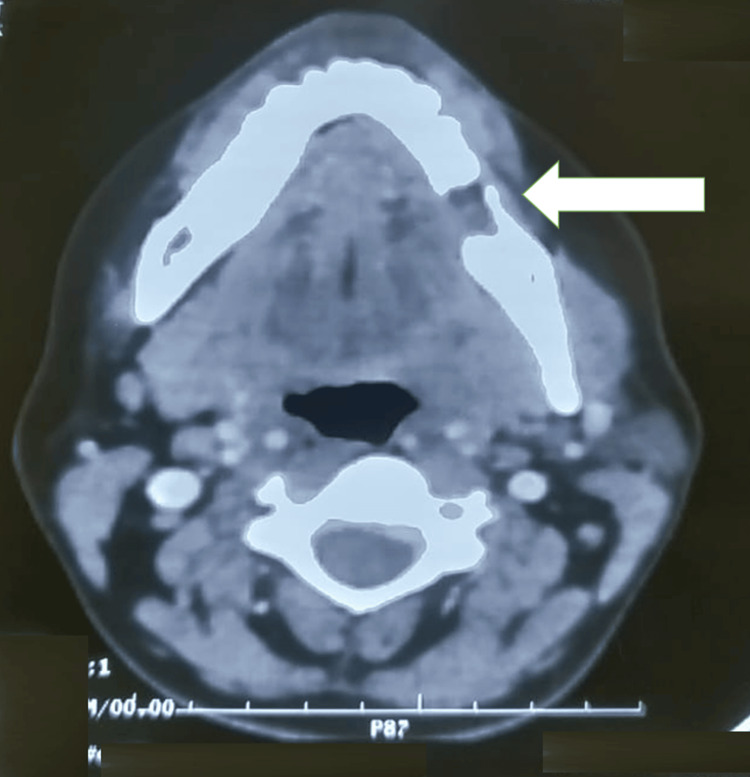
Contrast-enhanced CT (axial) of the oral cavity showing a 10 x 8 mm soft tissue lesion in the edentulous socket of the left mandibular first molar (arrow).

**Figure 3:**
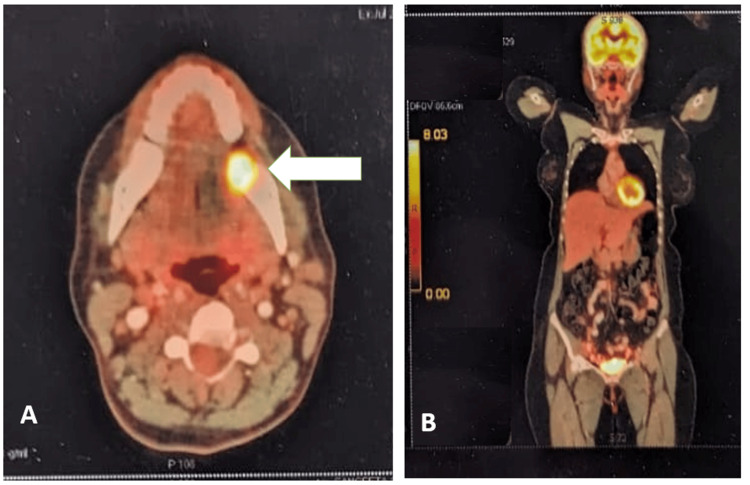
PET-CT scan demonstrating a metabolically active lesion at the primary site (SUVmax-17.6), with no evidence of abnormal metabolic activity elsewhere in the body. PET: positron emission tomography.

Anticipating a malignant lesion, an incisional biopsy was performed under local anesthesia. It revealed atypical melanocytes in the vertical growth phase, exhibiting cellular and nuclear pleomorphism, and an altered nuclear-cytoplasmic (NC) ratio. Long spindled cells with nuclear hyperchromasia and dense eosinophilic cytoplasm were also seen. Another feature noted was the frequent mitosis and cytoplasmic brown pigment in spindle cell melanoma. Hence, the diagnosis of malignant melanoma was established (Figures 4A, 4B).

**Figure 4:**
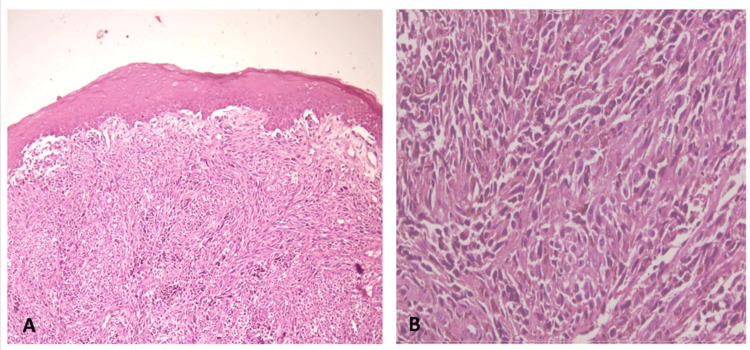
(A) and (B) Histopathology of the oral melanotic lesion. (A) Mucosa formed of stratified squamous epithelium. Subepithelium expanded by spindled cells with nuclear pleomorphism and cytoplasmic brown pigment in many cells (hematoxylin & eosin x200). (B) Long spindled cells with nuclear hyperchromasia and dense eosinophilic cytoplasm and cytoplasmic brown pigment in spindle cell melanoma. Frequent mitosis was also noted (hematoxylin & eosin x600).

Given the aggressive nature of the disease in the reported literature, the patient was taken up for wide local excision with segmental mandibulectomy and prophylactic modified radical neck dissection (MRND) type 2 (Figure 5). Intra-operatively, tissue from the resected margins was sent for frozen-section analysis to confirm negative margins (all margins free; the closest was the medial mucosal margin, 0.5 cm away and free of tumor). The resultant defect was closed with a pectoralis major myocutaneous (PMMC) flap, primarily with the aim of restoring soft tissue bulk and achieving adequate wound coverage in the immediate postoperative period. The primary goal for this patient was functional rehabilitation (maintenance of oral competence, speech, and swallowing). Given the aggressive nature of OMM and the need for close oncologic monitoring, the treatment approach was focused on disease control and functional restoration, rather than definitive rehabilitation with dental implants.

**Figure 5:**
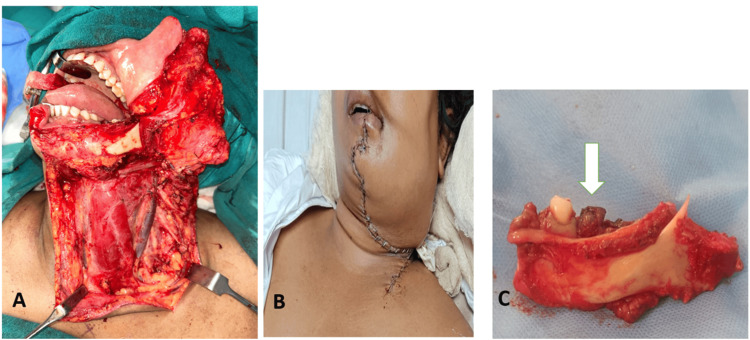
(A)-(C) Surgical management and resected specimen of oral malignant melanoma. (A) and (B) Intraoperative views following wide excision of primary lesion with segmental mandibulectomy and modified neck dissection. (C) Resected specimen demonstrating the melanocytic lesion (arrow).

Postoperative histopathological examination confirmed malignant melanoma, and all the resected lymph nodes were negative for metastasis. Adjuvant radiotherapy was planned; however, the patient defaulted early and received only 20 Gy in 11 fractions. She did not complete the planned course of adjuvant radiation owing to severe mucositis, odynophagia, and logistical constraints. Currently, the patient remains on regular three-monthly follow-up and is progressing well, with a well-healed primary site and satisfactory integration of the PMMC flap (Figure 6). Despite suboptimal completion of the intended regimen, she continues to be disease-free at 18-month follow-up, without any clinical evidence of recurrence.

**Figure 6:**
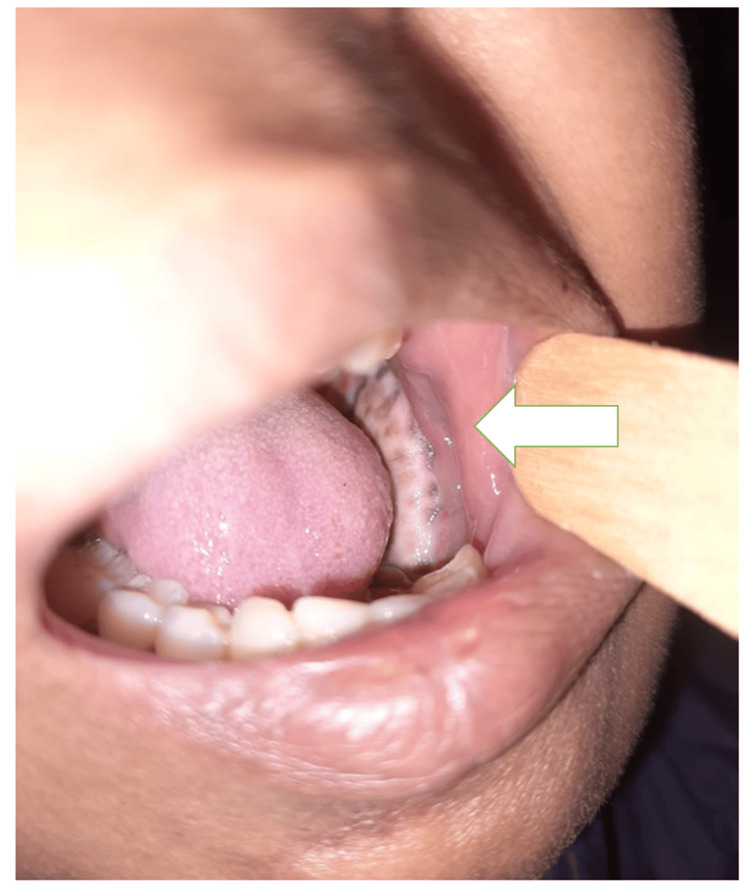
One-month post-surgery, satisfactory uptake of the PMMC flap at the primary site. PMMC: pectoralis major myocutaneous.

## Discussion

Liversedge quoted, ‘Oral malignant melanoma is a lesion of such rarity that most clinicians are unlikely to see more than a few cases in their practicing career’ [5]. It is a highly aggressive tumor with a poor prognosis, largely due to late detection and early locoregional and distant metastasis. Lungs and liver are the usual sites for distant metastasis. In addition to the oral mucosa, malignant melanoma can involve other mucosal sites, including the nasal cavity and paranasal sinuses, pharynx, and conjunctiva. Mandibular involvement, as in the present case, is extremely uncommon.

The oral lesion may appear mucosal pink to brown, bluish, or even black, with or without ulceration, and is usually painful. The differentials for a melanotic lesion in the oral cavity include dental amalgam, smoking-induced melanosis, melanotic macule, drug-induced pigmentation, Peutz-Jeghers syndrome, and even Addison's disease. Dental amalgam presents as a localized blue-gray macule adjacent to a restored tooth. Apart from an obvious history of prior dental procedure, clinically an amalgam appears well-defined and non-progressive. Radiologically, it may demonstrate fine metallic radiopaque particles without any associated bone destruction. A local focus of hemorrhage may appear similar, especially in elderly patients on anticoagulants. A melanotic macule, however, occurs secondary to increased melanin production by the existing melanocytes. There is no actual proliferation of melanocytes (unlike melanoma), and it appears as a small, well-circumscribed, flat lesion (macule). There is no associated bone destruction or specific radiological findings. Kaposi sarcoma, a rare neoplasm associated with HIV infection, may also appear as a pigmented lesion in the oral cavity. Even in the present case, the lesion appeared bluish without any surrounding ulceration and closely resembled a hematoma on initial examination. Hence, a high index of suspicion is required to catch the diagnosis.

To consider a lesion as oral malignant melanoma, Greene et al. in 1953 suggested the following criteria: (1) Demonstration of both clinical and histological evidence of malignancy in the oral mucosa, (2) Evidence of junctional activity (i.e., melanocytes arranged along the basal layer of the surface epithelium) in the lesion, and (3) Inability to demonstrate melanoma at any other primary site [6]. Our patient fulfilled this criterion. However, the presence of ‘junctional activity’ is now regarded as an unreliable indicator of a primary lesion because metastatic melanoma can similarly involve the epithelial junction.

Oral malignant melanoma may either arise within a benign melanocytic lesion or de novo from melanocytes within otherwise healthy mucosa. While ultraviolet radiation drives skin melanoma, the oral variant is linked to tobacco use, formaldehyde exposure, and dental irradiation. The presence of oral melanosis also predisposes to its development. OMM is reported to have a more aggressive course than cutaneous melanoma [7]. In 2010, the American Joint Committee on Cancer adopted a staging system for head and neck mucosal melanoma. There are no stages 1 or 2; they started at stage 3 for mucosal disease and at stage 4 for moderate and advanced disease [8]. This reflects the aggressive nature of the condition. Interestingly, oral melanoma is seen to be more common in Japanese individuals and those with darker skin tones, although it can occur in patients of all racial backgrounds [9]. Takagi et al. in 1974 reported a large series of 120 cases of primary melanoma of the oral cavity in the Japanese population and reported a higher predisposition in male patients and in those of Japanese descent [10]. However, the present case describes a woman patient from India. The mean age of onset is 55 years (40-70 years) [11]. In the present report, the patient was in her forties, consistent with the findings described by Smyth et al. [11]. However, she had no history of prior irradiation or tobacco use.

The diagnosis of oral malignant melanoma is challenging, requiring a high degree of suspicion upon encountering a focal oral pigmented lesion. Biopsy and histopathological examination are essential for confirming the diagnosis. Histologically, OMM is typically composed of atypical melanocytes that invade the underlying tissue, with varying degrees of pigmentation. Immunohistochemical staining for markers such as homatropine methylbromide (HMB-45), S100 protein, and Melan-A can assist in differentiating melanoma from other malignancies or benign lesions [3].

Umeda and Shimada in 1994 proposed a management protocol for OMM, suggesting a wide intraoral excision with 1.5cm margins, excision of any lymph nodal metastasis, and consideration of adjuvant chemotherapy [12]. To date, the treatment philosophy still involves wide local excision with clear margins, with surgical resection remaining the most effective approach for localized disease. Elective neck dissection is advocated [13]. Due to the aggressive nature of OMM, recurrence is common, and patients often require adjuvant radiotherapy to reduce the risk of local recurrence and metastasis [14]. However, despite incomplete adjuvant radiotherapy, the present patient has maintained adequate locoregional control on follow-up. Owing to its complex anatomic location and proximity to vital structures, achieving clear margins during OMM resection can be difficult. Positive surgical margins are seen in up to 43% of cases and are associated with poor outcomes [15].

Local disease failure serves as a harbinger for distant metastasis because most patients who succumb to distant metastasis also have local or regional recurrences. Therefore, the addition of radiotherapy for locoregional control should be considered [12]. In addition, radiation therapy is also advocated for unresectable lesions and palliation, even though melanomas are not traditionally considered to be radiosensitive tumors [16]. In advanced cases, chemotherapy or immunotherapy may be considered, although the data on their efficacy continues to evolve.

Targeted systemic therapies have recently shown promise in the management of metastatic oral melanoma. Particularly, in 2011, the first immune checkpoint inhibitor against human cytotoxic T-lymphocyte antigen 4 (CTLA-4), ipilimumab, was approved for advanced inoperable metastatic disease [17]. However, the anti-programmed cell death protein 1 (PD-1) monoclonal antibody pembrolizumab (KEYNOTE-006 trial) demonstrated better progression-free survival and fewer side effects than ipilimumab and has now been approved as first-line therapy for unresectable or metastatic disease [18]. Another PD-1 inhibitor, nivolumab, has shown better efficacy and fewer side effects than ipilimumab, and has been approved for adjuvant therapy (CheckMate 238 trial) [19]. In addition, ipilimumab and nivolumab have shown synergistic effects on the immune response against advanced melanoma [20]. In March 2022, a fixed-dose combination of relatlimab (an anti-LAG-3 monoclonal antibody) with nivolumab (RELATIVITY-047 trial) demonstrated improved efficacy compared with nivolumab monotherapy and received FDA approval for the management of metastatic or unresectable melanoma [21].

The overall prognosis for oral melanoma remains guarded, with survival rates significantly lower than those for cutaneous melanoma, which highlights a persistent failure in early detection. The presence of lymph nodal metastasis serves as the most critical indicator of a poor outcome [22]. The five-year survival rate, as reported by Shah et al., is 25% for node-negative patients and 19% for node-positive patients [23]. The overall mean survival for OMM is reported to be around 28 months [24]. Early detection remains a key factor in improving patient outcomes, but given the disease's rarity and the lack of specific symptoms, timely diagnosis remains a significant challenge.

## Conclusions

In conclusion, oral malignant melanoma (OMM) presents a significant clinical challenge due to its rapidly progressive nature and the diagnostic difficulties it poses for practitioners. Involvement of the mandible or the gingiva is an uncommon presentation. Especially in the early stages, this malignancy frequently mimics the more common, benign oral conditions, which can lead to critical delays in management. Clinicians must maintain a high index of suspicion when evaluating any pigmented oral lesion, with a low threshold for biopsy. We wish to emphasize that thorough oral screenings must be undertaken regularly wherever possible, as early identification remains the most critical factor in improving survival.

The aggressiveness and overall rarity of the disease necessitate a multidisciplinary approach to care. The treatment guidelines continue to evolve, and even with the addition of chemotherapy and immunotherapy, the long-term outlook for patients remains concerning at present. The patient in the present report could not complete the planned radiotherapy. Despite this, she has remained disease-free for a considerable period. However, this outcome should be viewed with caution. It is likely a one-off observation and cannot be generalized. Given the aggressive disease biology, completing the prescribed treatment is essential. Patients should be strongly encouraged to adhere to the full course of the therapy. The patient should be kept under close, long-term follow-up due to the risk of late recurrence.
